# An Immature Ten-year Long-standing Case of Ossifying Fibroma

**DOI:** 10.7759/cureus.2782

**Published:** 2018-06-11

**Authors:** Shaimaa M Abu el Sadat, Mohamed K Al Ashiry, Raghdaa A Mostafa, Ahmed Z Abdelkarim, Ali Z Syed

**Affiliations:** 1 Oral Radiology Department, Ain-Shams University, Cairo, EGY; 2 Department of Anatomy, Biochemistry & Physiology, University of Hawaii School of Medicine, Honolulu, USA; 3 Department of Oral Medicine and Diagnostic Sciences, CWRU School of Dental Medicine, Cleveland, USA

**Keywords:** high resolution 3d cbct-based imaging, ossifying fibroma, panoramic, fibrous osseous lesion

## Abstract

Ossifying fibroma is a rare benign bone neoplasm common in middle age, with definite female predominance. Here, we describe a case of an ossifying fibroma in a 36-year-old female, with a right facial deformity. The lesion had been present for almost 10 years. The panoramic image showed a multilocular appearance with scattered radiopacities. Advanced imaging revealed an expansile multilocular lesion with multiple small radiopaque foci and a few dense radiopaque masses. A histopathological examination confirmed the diagnosis. The case represents a non-aggressive form of an immature ossifying fibroma.

## Introduction

Cemento-ossifying fibroma (COF) is classified as, and behaves like, a benign bone neoplasm. It is often considered a type of fibro-osseous lesion (FOL) [1]. These FOL lesions represent a complex and diverse group of lesions characterized by a nearly similar histological appearance [2]. In FOL lesions, the normal bone is replaced with fibroblastic stroma containing varying amounts of abnormal bone and/or cementum-like tissue [2].

Fibro-osseous lesions are benign lesions of a heterogeneous group with an unknown etiology, affecting the maxilla, mandible, and other craniofacial bones [3]. Lesions in this category include focal cemento-osseous dysplasia, COF, and fibrous dysplasia [4]. This group often presents with a similar clinical presentation, radiographic appearance, and histological criteria. It, therefore, poses a difficulty in diagnosis, classification, and management for the clinician [5].

The COF presents radiographically as a well-defined, either unilocular or multilocular lesion with smooth contours [3]. The degree of radiopacity depends on the maturity of the lesion. The mature lesion may appear entirely radiopaque whereas the immature lesions may present as a complete radiolucent lesion. However, other lesions can present with varying degrees of radiolucency [3,6]. In histology, COFs are well-circumscribed, occasionally encapsulated, consisting of cellular fibrous tissues with thin, isolated trabeculae of bones. The trabeculae may show osteoblastic rimming and spherical foci of calcified material, which are relatively acellular, resembling cementum [6].

In the past, COF lesions were classified as two separate entities, depending on the type of internal content. When the predominant calcified material was bone, the term ossifying fibroma was used. With predominantly cementum-like material, the term cementifying fibroma was used [6-7]. They were believed to have different origins; However, the term ‘‘cemento-ossifying fibroma’’ was replaced by ossifying fibroma in the new world health organization (WHO) classification in 2005 [1].

## Case presentation

A 36-year-old female presented to the oral diagnosis clinic at Ain Shams University's dental school for the evaluation of a swelling in the lower right quadrant. The patient reported the swelling to be of 10 years' duration with a progressive course during the last year. The patient reported some pain and difficulty during mastication. There was a history of surgical biopsy several years ago, but a biopsy report was not available.

**Figure 1:**
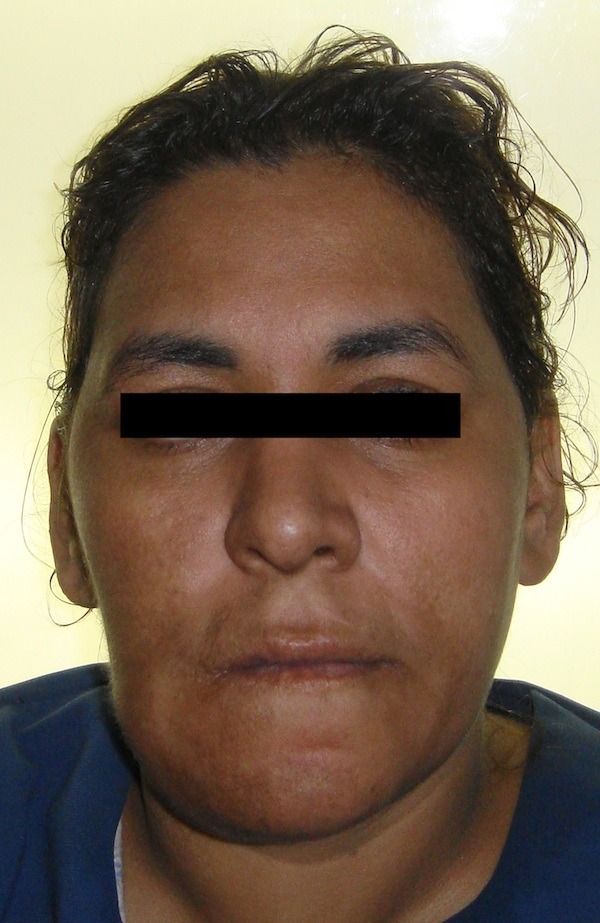
Extraoral examination shows right facial asymmetry

An extraoral examination revealed right facial asymmetry with normal overlying skin (Figure 1). Submandibular lymph nodes were palpable on both sides but were not tender. An intraoral examination showed a hard bony swelling related to the right mandibular premolar-molar region. On clinical examination, the mucosa appeared normal, with no evidence of ulceration or bleeding.

**Figure 2:**
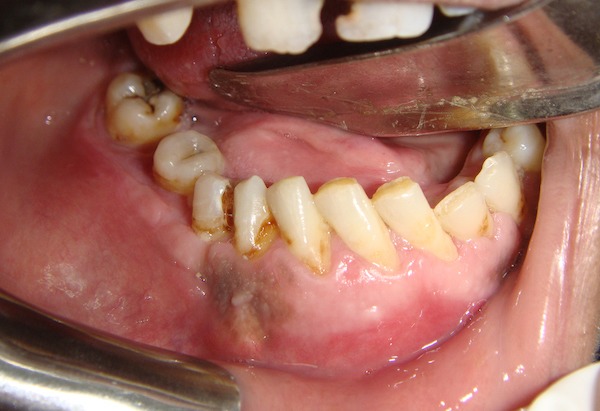
Intraoral photo reveals normal mucosa, with expansion and drifting of the teeth

A massive buccolingual expansion of the lesion was noted, possibly crossing the midline. The lesion was tender. There was no associated tooth mobility. However, drifting and displacement were noticed (Figure 2). The related teeth were also vital. Oral hygiene was poor. Medical history was unremarkable, with vital signs within normal range.

**Figure 3:**
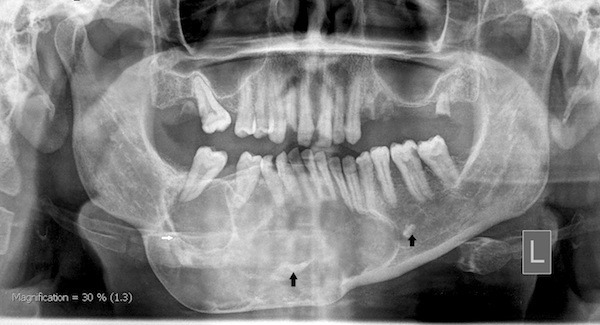
Panoramic radiograph showing an expansile multilocular lesion with radiopaque masses (arrows)

The panoramic radiograph (OP100, Instrumentarium Imaging, France, at kVp 66, 13 mA) showed an expansile multilocular radiolucent lesion involving the mandible. The lesion extended from the lower-right second molar to the contralateral second premolar crossing the midline. The borders were well demarcated and sclerotic. Superiorly, the lesion extended to the alveolar crest, causing expansion. Inferiorly, it spread to the inferior border, causing displacement. Extreme thinning and bowing of the inferior cortex of the mandible was evident but contained an intact cortex. The inferior alveolar canal on the right side appeared to be displaced inferiorly. The divergence of the roots of the related teeth showed a loss of the lamina dura, as well as evidence of slight root resorption in the lower right canine. Multiple radiopaque masses with density compared to that of bone were seen within the lesion (Figure 3).

**Figure 4:**
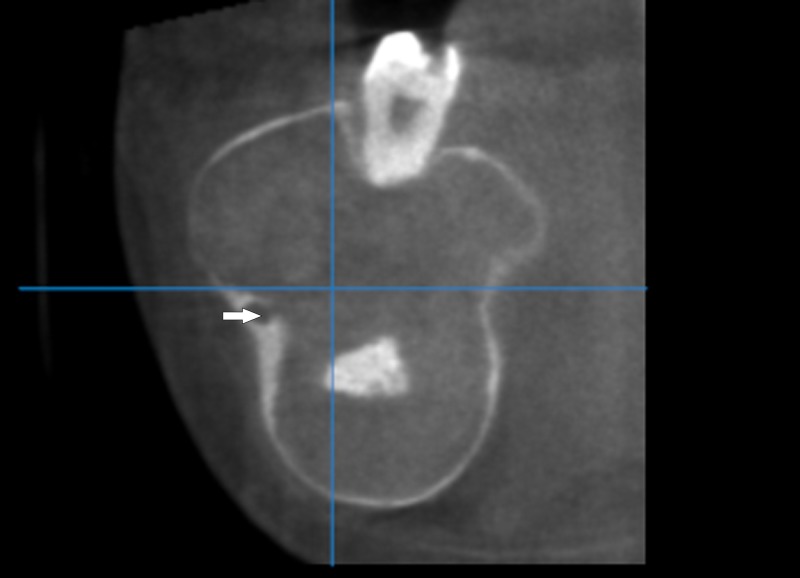
Coronal cut shows a buccolingual expansion of the lesion with the displacement of the mandibular canal (white arrow)

Advanced imaging cone beam computed tomography (CBCT), using Scanora 3D (Soredex, Wisconsin, USA; at 85 kVp, 15 mA, and 337mGy) with 3.5 mm slice thickness, revealed a lesion measuring 45.9 mm x71 mm. The CBCT images showed massive buccolingual expansion with extreme thinning of the cortical plates. The internal structure showed a multilocular appearance with fine septa. Some slices showed fine granular radiopaque foci, while others showed dense radiopaque masses. Although evidence of expansion was noted all over the lesion, the cortical plates appeared to be intact with no perforation (Figure 4).

The differential diagnosis includes other lesions, such as fibrous dysplasia, calcifying cystic odontogenic tumor (Gorlin cyst), calcifying epithelial odontogenic tumor, central giant cell granuloma, and adenomatoid odontogenic tumor. The well-defined border of the central cemento-ossifying fibroma helps to differentiate it from the aggressive mixed lesions as chondrosarcoma, osteosarcoma and metastatic malignant lesions [3,8].

Aspiration biopsy was negative. An open bone biopsy was performed via the intraoral approach, and multiple soft tissue and bone fragments were removed and submitted in 10% formalin for histological evaluation.

**Figure 5:**
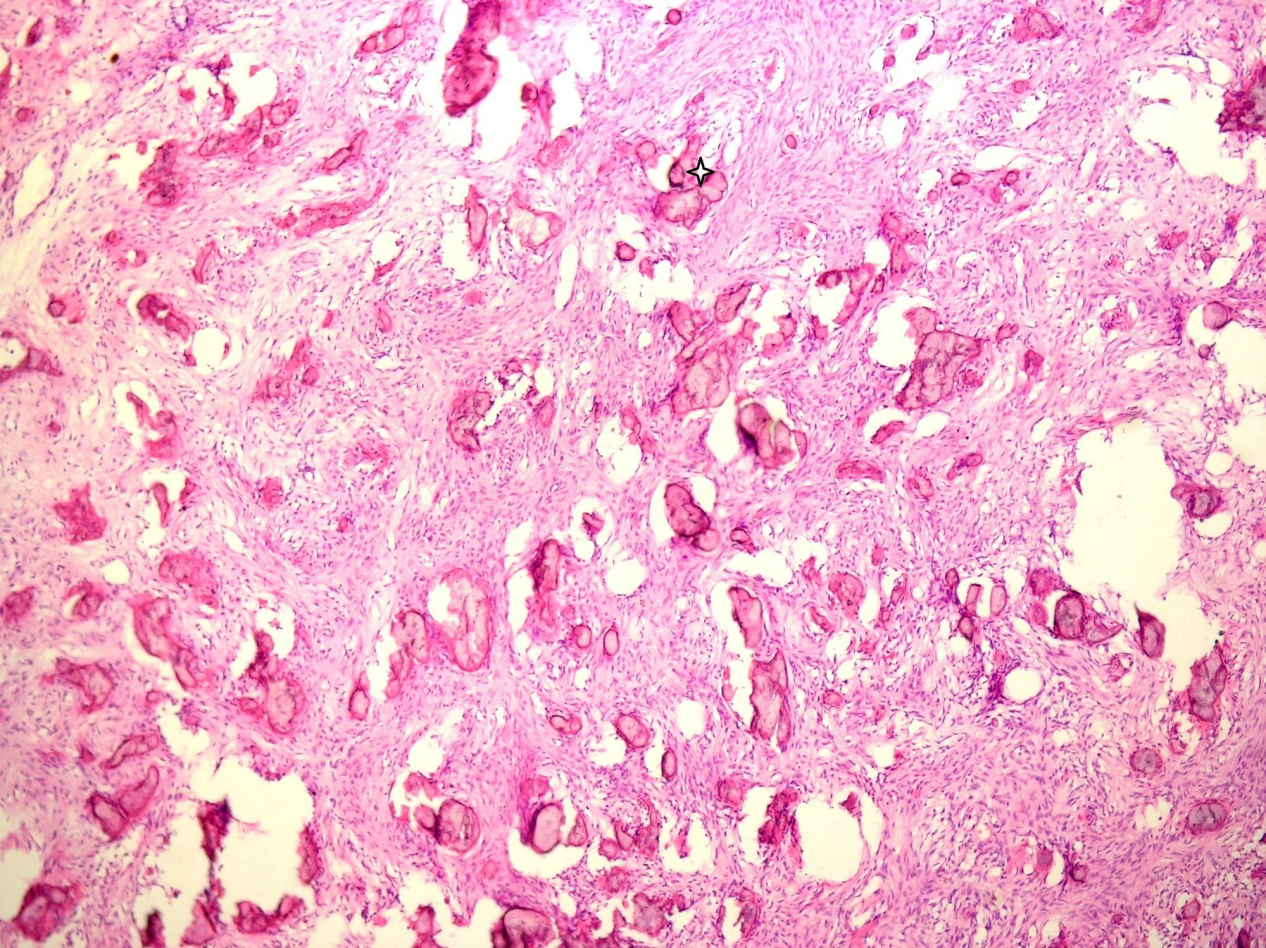
The histopathological picture. A highly cellular fibrovascular lesion with spindle fibroblasts forming a whorled pattern. Irregular bone trabeculae with osteoblastic rimming, dystrophic calcifications, and areas of cementum-like tissue were also noted (star)

The microscopic examination showed a highly cellular fibrovascular lesion with spindle fibroblasts forming a whorled pattern. Irregular bone trabeculae with osteoblastic rimming, dystrophic calcifications, and areas of cementum-like tissue were also noted (Figure 5). No signs of malignancy were observed. The histopathological features suggest ossifying fibroma. The patient was referred to the maxillofacial surgery department for lesion removal and jaw reconstruction.

## Discussion

Here, we report a case of COF with a long-standing history of 10 years. Fibro-osseous lesions are a heterogeneous group of benign lesions of unknown etiology affecting the jaws and other cranial bones. Lesions in this category include fibrous dysplasia (FD), cemento-osseous dysplasia (COD), and cemento-ossifying fibroma (COF) [9].

In 1992, WHO classified COF as a true bone neoplasm, and the term cemento-ossifying fibroma was replaced by ossifying fibroma in the WHO classification of 2005 [1]. Support for a neoplastic etiology includes examples of persistent, locally aggressive growth characteristics and recurrence in a few cases [1]. Chromosomal translocations have also been described in a few examples [5]. In some cases, the existence of previous trauma in the area has been established as a possible etiological agent [10].

It’s been previously suggested that the origin of this tumor is either odontogenic or from the periodontal ligament. However, identical neoplasms with the cementum-like material have been identified in the orbital, frontal, ethmoid, and sphenoid bones. This negates the previous theories [6].

Ossifying fibromas (OF) are more frequent in females than in males, with a female-to-male ratio as high as 5:1 [11]. Although the age of ossifying fibroma tends to be variable, it’s predominant in the third or fourth decades of life [3,4,12]. The mandible is the most commonly affected location with the premolar-molar area superior to the inferior alveolar canal the most frequent site. The lesion is generally asymptomatic although pain was reported in some cases. Clinically, the growth of the lesion produces a noticeable swelling and mild deformity [3,13]. Displacement of teeth is common; root resorption may also be seen. When the tumor arises in children, it’s called juvenile ossifying fibroma. This is a benign but potentially aggressive form of ossifying fibroma and occurs in the first two decades of life [12].

The above features were very consistent with our case. The patient suffered from facial deformity and gave a history of a slowly growing mass over a period of 10 years. The patient also reported some pain during mastication. The lesion caused tooth displacement without affecting their vitality, and the overlying mucosa remained intact.

The radiological features are variable depending on the stage of development. According to Eversole, two basic patterns of ossifying fibroma may be encountered: (1) The more prevalent unilocular radiolucency with or without radiopaque foci and (2) the multilocular radiolucent configuration [12]. In the early stages, ossifying fibroma appears as a well-defined unilocular or multilocular intra-osseous radiolucent lesion without evidence of radiopacities. As the tumor matures, there is increasing calcification so that the radiolucent area becomes flecked with opacities until, ultimately, the lesion appears as a radiopaque mass surrounded with a radiolucent rim [8].

The presented case showed a multilocular appearance with an internal structure varying from small radiopaque foci that were seen in the cross-sectional images. CBCT images revealed large dense radiopaque masses, which were evident in both a panoramic radiograph and CBCT. The multilocular configuration wasn’t widespread for ossifying fibroma. Although the lesion has been present for almost 10 years, it doesn’t appear in the mature form, i.e., a completely radiopaque mass surrounded with a radiolucent periphery. Thus, this case could be considered an immature form of an ossifying fibroma.

Regardless of the stage of development, the lesion is always well-circumscribed and well-demarcated. In this case, the radiographic borders of the tumor appeared to be relatively smooth, well-defined, and mostly corticated. Some areas of the lesion showed bone sclerosis. A marked expansion, displacement of the inferior alveolar canal downwards, and the bowing of the inferior cortex of the mandible were seen in the panoramic radiograph. These features characterize ossifying fibroma [3,8]. In the CBCT images, the lesion caused a massive expansion of the cortical plates. The significant point is that the outer cortical plate, although displaced and thinned, remained intact. One additional important feature that facilitated the diagnosis is that the lesion has a spiral growth pattern rather than a linear one, i.e., the lesion grows from a specific epicenter appearing to be growing equal in all directions and presents as a round tumor mass [1,3,8-9].

The radiologic differentiation of a central ossifying fibroma is challenging; however, it was included here in the differential diagnosis because of the characteristic centrifugal growth pattern. The final diagnosis was confirmed by the histologic appearance. In fibrous dysplasia, an intimate continuity is found between the lesion and the adjacent normal bone. The tumor expands throughout its length, and the margins are diffuse and radiographically poorly defined. Its radiological structure is more homogenous than that of ossifying fibroma, both of which appear with internal radiopacities. Ossifying fibroma is a well-circumscribed tumor that grows expansively, its boundaries are better defined, and these lesions occasionally have a soft tissue capsule and cortex [3-4]. The differentiation of both lesions is significant, as the management differs in each case.

Histopathologically, ossifying fibroma consists of fibrous tissue that exhibits varying degrees of cellularity and contains mineralized material. The hard tissue portion may be in the form of trabeculae of osteoid and bone or basophilic poorly cellular spherules that resemble cementum [6]. Here, the lesion showed highly cellular fibrous stroma and the fibroblasts showed a whorled pattern. Some areas showed trabeculae of bone with osteoblastic rimming areas of dystrophic calcification, which is explained by the long duration of the lesion. No malignant signs were seen. Cementum-like material was also observed, which facilitated the diagnosis as the ossifying benign neoplasm.

It is generally claimed that ossifying fibromas are lesions that are well-separated from the adjacent cancellous bone. They are encapsulated lesions that help to separate them from the surrounding bony bed and tend to be removed with ease during surgical treatment [5,14].

These lesions are treated by radical resection or conservatively by local excision with curettage. Most lesions are treated by enucleation, and recurrence is low. Slootweg and Müller suggested that there were no differences between the cases that have limited surgical treatment and those with significant surgery with respect to results, and they recommended conservative surgery. Sweet et al., however, did report a recurrence rate of almost 25% for maxillary lesions, but some of these may have been examples of juvenile active ossifying fibromas, which are more aggressive than ossifying fibroma [15-16]. There is no evidence of malignant transformation in these cases [17].

## Conclusions

Fibro-osseous lesions are benign lesions of a heterogeneous group with a broad spectrum of clinical, radiographic features. COF lesions can be very slow growing. Symptoms are often gradual and long-standing in some cases. Follow-up is required for these cases.
